# Clinical applications of artificial intelligence in robotic surgery

**DOI:** 10.1007/s11701-024-01867-0

**Published:** 2024-03-01

**Authors:** J. Everett Knudsen, Umar Ghaffar, Runzhuo Ma, Andrew J. Hung

**Affiliations:** 1 Keck School of Medicine, University of Southern California, Los Angeles, USA; 2 Cedars-Sinai Medical Center, Los Angeles, USA

**Keywords:** artificial intelligence, robotic surgery, robot-assisted surgery, intraoperative enhancement, clinical improvement, ethical considerations of AI

## Abstract

Artificial intelligence (AI) is revolutionizing nearly every aspect of modern life. In the medical field, robotic surgery is the sector with some of the most innovative and impactful advancements. In this narrative review, we outline recent contributions of AI to the field of robotic surgery with a particular focus on intraoperative enhancement. AI modeling is allowing surgeons to have advanced intraoperative metrics such as force and tactile measurements, enhanced detection of positive surgical margins, and even allowing for the complete automation of certain steps in surgical procedures. AI is also Query revolutionizing the field of surgical education. AI modeling applied to intraoperative surgical video feeds and instrument kinematics data is allowing for the generation of automated skills assessments. AI also shows promise for the generation and delivery of highly specialized intraoperative surgical feedback for training surgeons. Although the adoption and integration of AI show promise in robotic surgery, it raises important, complex ethical questions. Frameworks for thinking through ethical dilemmas raised by AI are outlined in this review. AI enhancements in robotic surgery is some of the most groundbreaking research happening today, and the studies outlined in this review represent some of the most exciting innovations in recent years.

## Introduction

Since its inception and widespread adoption, artificial intelligence (AI) has revolutionized nearly every aspect of human life. AI is the study and development of algorithms that give machines the ability to reason and perform cognitive functions such as problem-solving and decision-making [1]. From finance to agriculture, manufacturing to education, AI has fundamentally altered our ability to understand and respond to complex problems. Perhaps the most impactful adoption of AI on human life is in the field of medicine where AI is being used to help physicians make more precise decisions and predict patient outcomes with a higher degree of certainty. Within the medical field, surgery has experienced one of the largest impacts with the adoption of AI as more and more surgeries are performed using robotic assistance. Current surgical robots are controlled by a “master–slave” dynamic where the robot itself does not have any autonomy if it does not have a human operator. However, recent advances in AI and machine learning (ML) seek to expand the capabilities of surgical robots and augment the surgical experience in the operating room. Surgical robots rely on data captured through sensors and images to operate, and this plethora of data capture is the key driver behind AI innovations in robotic surgery [1].

**Fig. 1:**
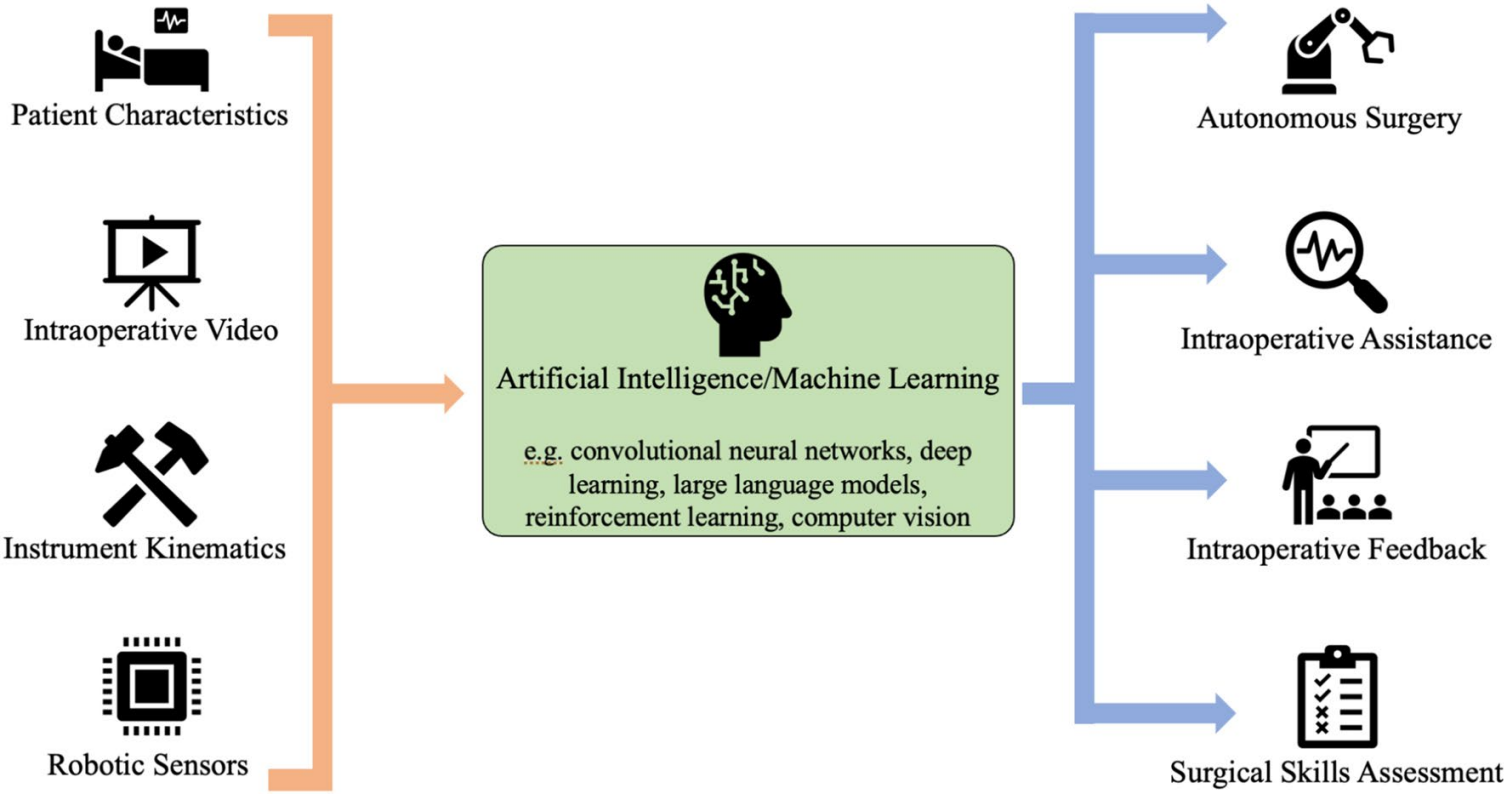
Data inputs and outputs for the development of artificial intelligence/machine learning applications for the improvement of robotic surgery

In this review, we focus on the recent advancements that AI brings to the world of robotic surgery with a particular emphasis on intraoperative applications. We also outline important ethical considerations for the incorporation of AI into robotic operations. Broadly, intraoperative enhancements provided by AI can be classified into two categories: robotic autonomy and surgical assessment/feedback. Advances in each of these categories are focused on creating environments for safe, data-informed surgical decision-making and enhancing surgical education (Fig. 1). Robotic surgery’s continued integration of AI will improve patient outcomes and make surgery safer in the years to come.

## Methods

**Fig. 2:**
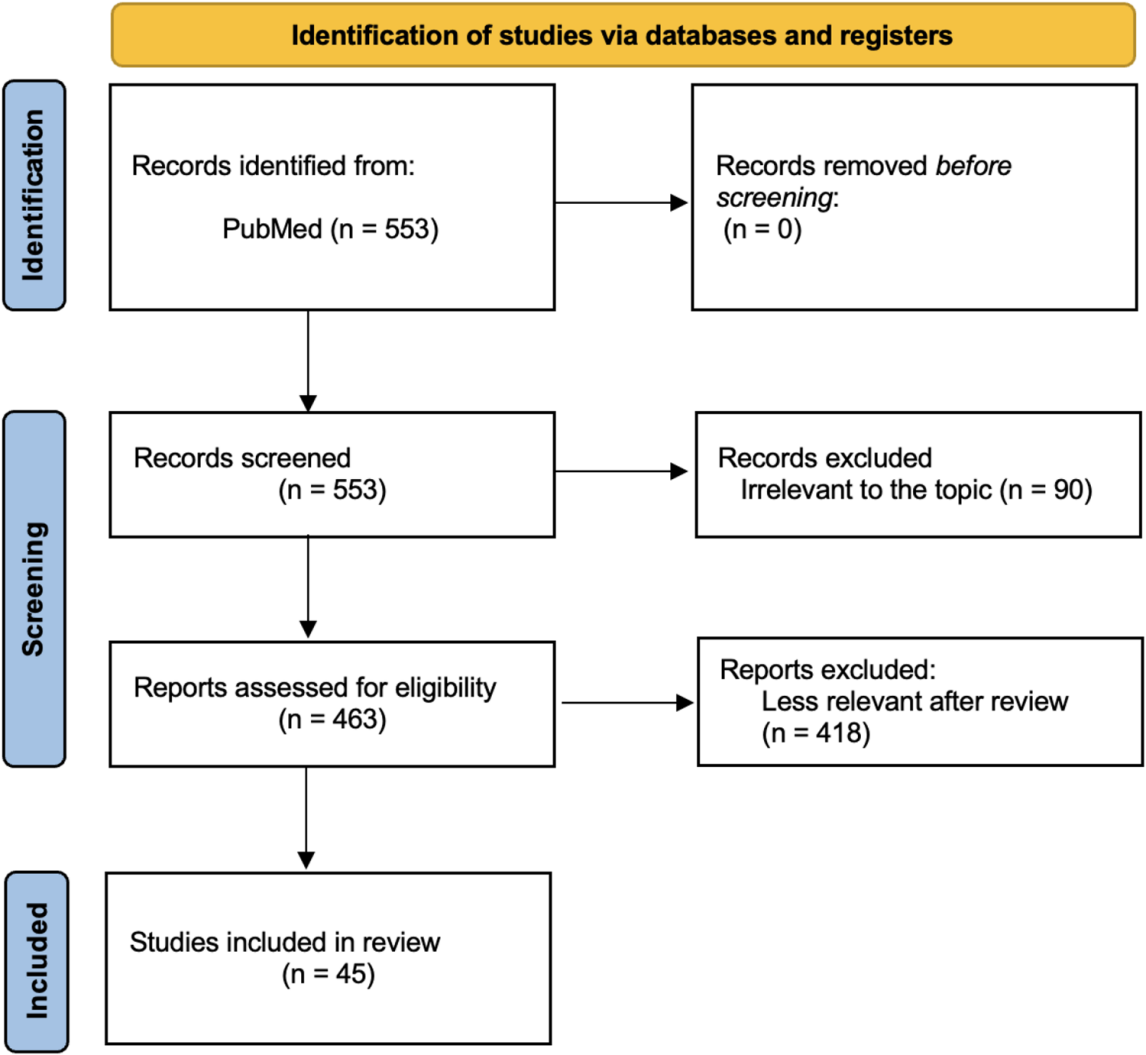
PRISMA flow diagram for literature selection. PRISMA   preferred reporting items for systematic reviews and meta-analyses

A literature search for this narrative review was completed using the PubMed database. Literature was limited to 2 years (November 2021–November 2023) to select papers representing the most recent advancements in the field. The search terms were as follows: (((((Artificial Intelligence) OR (Machine Learning)) AND (Robotic)) OR (Robot-Assisted)) AND (Surgery)) AND (Autonomy); (((((Artificial Intelligence) OR (Machine Learning)) AND (Robotic)) OR (Robot-Assisted)) AND (Surgery)) AND (Skill Assessment); and (((((Artificial Intelligence) OR (Machine Learning)) AND (Robotic)) OR (Robot-Assisted)) AND (Surgery)) AND (Feedback). For the context of this review, “robot” refers to a device that is assistive in the operating room and “artificial intelligence/machine learning” refers to the development of algorithms that give machines decision-making capacity [2]. The study selection process is outlined in the PRISMA flow diagram (Fig. 2). A total of 553 unique records were identified. Of these, 463 full-text articles were assessed for eligibility and 45 were selected as the most recent advancements in the field and are included in this narrative review.

## AI in intelligent assistance and robotic autonomy

The most widely used robotic surgical system today is the da Vinci (Intuitive Surgical, Sunnyvale, CA) which employs a “master–slave” relationship where a human surgeon performs all surgical gestures in a console setting. These gestures are then transmitted to the robotic arms docked in the patient surgical site. In other words, the da Vinci robot cannot act autonomously without the input of a human operator. Autonomy, however, is not a binary. Instead, autonomy can be thought of as a range from not autonomous all the way to completely autonomous, and the definitions of the levels of robotic surgical autonomy are laid out in Table 1. [3, 4] This section will highlight important advances in intraoperative robotic autonomy and assistance which are helping surgeons perform surgical tasks. This review explores innovations across all surgical subspecialties utilizing surgical robots.

**Table 1:** Levels of automation in robotic surgery. [3, 4] Adapted from Panesar et al. and Attanasio et al.

Human vs. Autonomous Robotic Surgeon		
Level 0 No Automation Traditional surgery: Human performs all surgical tasks; includes open, laparoscopic, and “master–slave” robotic surgical methods	Level 1 Some Assistance e.g. Intraoperative image guidance, augmented reality rendering; human surgeon still performs all surgical tasks	Level 2 Partial Automation Reduced required level of human input, but human surgeon still performs majority of surgical tasks
Level 3 Conditional Automation e.g. Automated bone drilling to prespecified depth with human setup; robot can perform certain procedural steps without human input	Level 4 High Automation e.g. Automated skin closure or lymph node dissection; Robot capable of performing most, if not all parts of a complex procedure with minimal human input	Level 5 Complete Automation e.g. Surgical robot that could perform an urgent cholecystectomy during a space flight; Robot is making all surgical decisions with no human input

### Surgical field enhancement

Robotic surgery allows for operation in deep anatomical spaces (e.g. abdominal and pelvic cavities, synovial joints) using small incisions for cameras and instruments. Real-time AI image enhancement (autonomy level 1) allows for enhanced identification of anatomical structures and instruments. Intraoperative visual environments are constantly changing as dissection or repair tasks progress which can lead to marked changes in intraoperative image quality. To combat these changes, Ali et al. have developed an online preprocessing framework capable of denoising, deblurring, and color-correcting real-time camera imaging to enhance intraoperative visualization in knee arthroscopy. Their method outperformed existing image enhancement with significantly reduced computation time to image display [5]. Robotic surgeons also often utilize electrocautery devices for dissection and ligation tasks, but this process generates smoke which remains trapped in the anatomical space of interest which can temporarily obscure the visual field until suction removal. Wang et al. have proposed a convolutional neural network (CNN) coupled with a Swim transformer that is capable of removing smoke from intraoperative surgical footage, ultimately producing an enhanced, smoke-free surgical view [6]. This is crucial when a surgeon needs to move quickly with good visualization such as during an acute intraoperative hemorrhage.

### Native tissue recognition

Beyond enhancing the surgeon’s intraoperative view, AI is also being used to provide intraoperative information on native tissue. Surgery often involves identifying “surgical planes” which are anatomical points at which tissues meet that are free of critical structures such as arteries, large veins, or nerves and are, therefore, safe for dissection. Kumazu et al. have developed a deep learning model using surgical video from robot-assisted gastrectomy capable of automatically segmenting loose connective tissue fibers to define a safe dissection plane (Fig. 2). Expert surgeons gave the model a mean sensitivity score of 3.52/4.00, indicating good model performance for safe plane identification. [7]

Another area of interest where AI promises advancement is surgical oncology, particularly in the realm of intraoperative positive margin minimization to prevent cancer recurrence. In the field of oral and oropharyngeal surgery, Marsden et al. presented a variety of AI models that utilize fiber-based fluorescence lifetime imaging to guide intraoperative dissection tasks. Model features allowed researchers to generate and overlay a heatmap of probable cancer location (ROC-AUC: 0.88) within the oral cavity to guide surgeons during cancer excision (Fig. 2) [8]. A second innovation in the field of neurosurgery utilizes data derived from an ultrasonic aspirator, a device commonly used to remove brain tumors. Bockelmann et al. generated AI models trained on brain tissue signal feedback from an ultrasonic aspirator. Models were able to distinguish signal differences between native tissue and brain tumor, achieving a mean F1 of 0.900 using a neural network approach. Intraoperative deployment of these tissue models can help surgeons to resect malignancies while preserving as much healthy neural tissue as possible [9]. A final example of surgical margin management was presented by Bianchi et al. in which they used preoperative multiparametric magnetic resonance imaging (mpMRI) to guide intraoperative frozen tissue sampling for margin detection during robot-assisted radical prostatectomy. An augmented reality 3D (AR3D) model was generated and projected onto the surgical field in the robotic console which identified the best location to take a frozen tissue specimen which, in theory, was free of cancer cells. Positive surgical margins were significantly lower at the level of the identified index lesion as compared to the standard non-AR3D approach (*p* = 0.01). [10]

### Instrument delineation

A surgical field is comprised of two major elements which are the native tissue (e.g. anatomical structures, malignancies) and non-native devices (e.g. surgical instruments, clips, sutures), and one of the greatest challenges in the implementation of AI in surgery is distinguishing between the two. Accurate delineation is critically important for the development of augmented reality ([AR] e.g., surgical field images with computer-generated overlay) surgical field enhancements. De Backer et al. presented a model consisting of deep learning networks to delineate instruments during robot-assisted kidney transplantation which achieved a Dice score of 97.10%. This is a marked improvement from the current standard in AR-guided robotic surgery with minimal disruption of 3D overlays highlighting key anatomical structures [11]. Ping et al. [12] presented a similar innovation for instrument detection for surgical endoscopy using a modified CNN and You Only Look Once v3 algorithm with sensitivity measurements of 93.02% and 87.05% for surgical instrument and tooltip detection, respectively [12].

### Tactile feedback

**Fig. 3:**
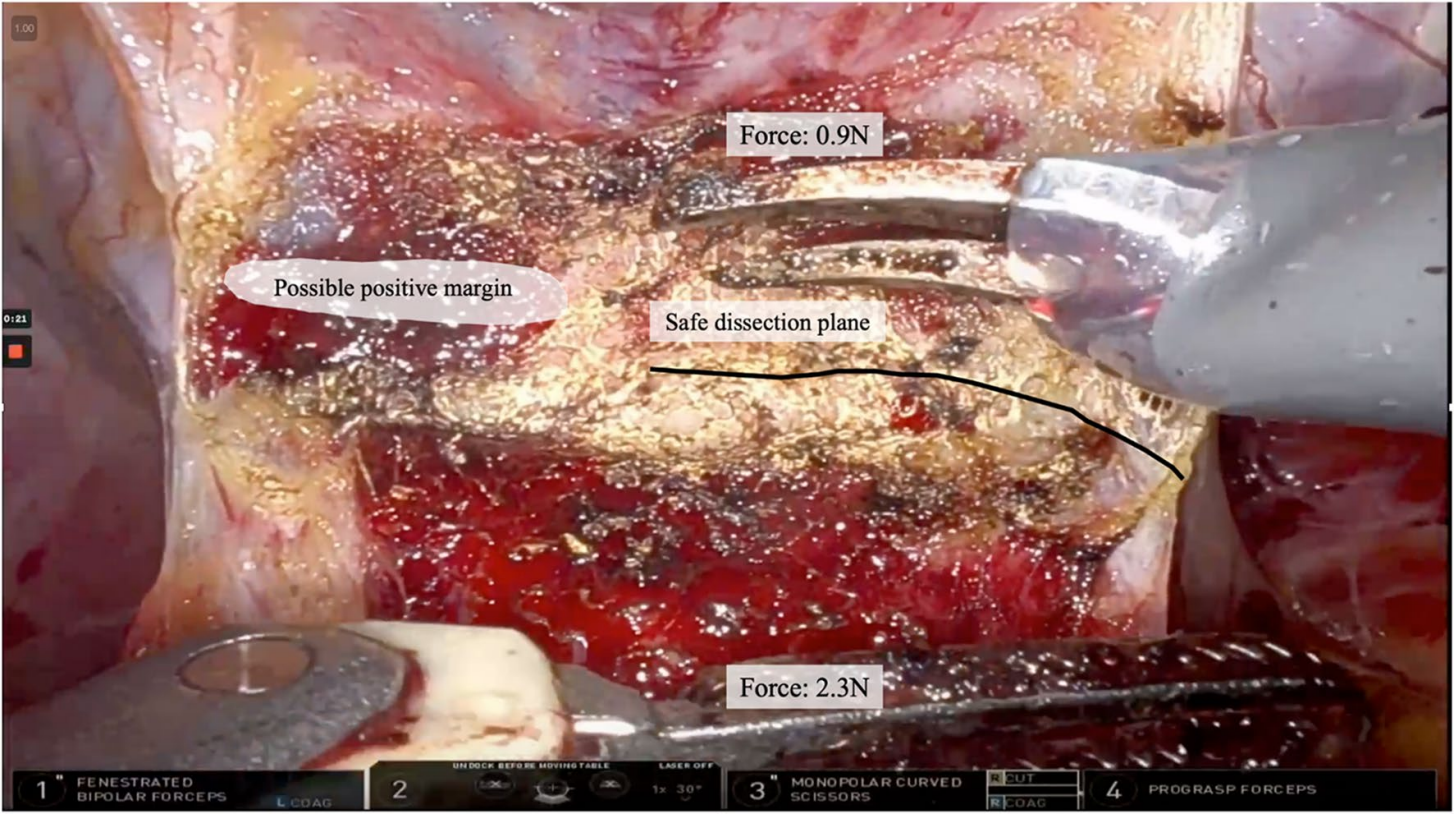
Potential console view showcasing AI-based intraoperative surgical field enhancements

One of the major differences between open and robot-assisted surgery is tactile sensation. Open approaches allow surgeons to palpate anatomical structures or feel changes in tissue resistance during dissection and suturing tasks, something that has not yet been developed for robotic surgery. The newest generation of surgical robots can display force measurements in the surgical console, but surgeons often struggle to understand how this value translates to intraoperative tissue force. To combat this, Miller et al. developed a study that provided surgeons with haptic (vibratory) feedback during an exercise in which surgeons were asked to draw a circle on a piece of paper with a surgical robot using as little force as possible. They found that the addition of haptic feedback reduced the median maximum exerted force from 6.43 N to 3.57 N (*p* < 0.001). In another paper, Doria et al. sought to apply haptic feedback to the palpation of anatomical structures. They first developed stiffness models to characterize the mechanical properties of intrauterine leiomyomas; they then adapted the stiffness models to deliver haptic feedback through a wearable fabric haptic device such that a greater vibration indicated stiffer tissue [13]. Force is also generated intraoperatively when retracting tissue, and excessive force can lead to preventable adverse events such as tissue tears or hemorrhage. Zhu et al. developed a robot for transoral surgery that uses piezoelectric sensors embedded in the instrument tips to detect forces on tissue up to 15 N. Sensed forces can then be displayed to the surgeon in the console, and automated warnings to surgeons can help prevent exceeding safe retraction forces (Fig. 3) [14].

### Stepwise automation

Final innovations in this section will focus on using AI to offload some of the physical and mental workload intraoperatively by the automation of certain surgical tasks. The first of these is the development of an algorithm for autonomous camera positioning on the da Vinci surgical robot. Eslamian et al. [15] developed a model that incorporates intraoperative tool tracking, robotic kinematics data, and intraoperative image data which is capable of autonomously translating the camera view and determining the correct zoom for optimal surgical visualization [15]. Suturing on the surgical robot is also seeing steps towards automation (automation level 2). Marques Marinho et al. presented a method that automates the key looping step required to suture an anastomosis during neonatal tracheoesophageal fistula repair. AI models generate intraoperative constraints (reduced degrees of freedom during instrument movement) to guide surgeons through the looping step of suturing. Surgeons reported decreased physical demand and shorter task duration using AI-guided suturing (both *p* < 0.05) [16]. Going beyond the automation of a single step in suturing, Saeidi et al. presented a study in which they developed a robotic system capable of fully automated laparoscopic bowel anastomoses. They developed a CNN coupled with a U-Net algorithm to determine the critical start and end positions for bowel anastomosis. The model then autonomously positioned a motorized Endo 360 suturing tool affixed to a surgical robot to perform the entire suturing task [17]. This is an example of autonomy level 4, but only for a very specific suturing task. However, this is still a marked step forward in the field of AI-enhanced robotic surgery with many more exciting innovations on the horizon.

## AI in robotic surgical assessment and feedback

### Workflow recognition

Automatic surgical workflow recognition (SWR) is an integral part of surgical assessment. A surgical procedure can be decomposed into activities at different levels of granularity including phases, steps, tasks, and actions [18]. Phases represent the overarching stages of a surgical procedure (e.g., access, execution of surgical objectives, and closure). Steps break down each phase into specific segments that contribute to the overall procedure (e.g., a nerve-sparing step of radical prostatectomy). Tasks are sub-components of a step (e.g., dissect and clip prostatic pedicles). Action is as specific as an individual motion carried out by a surgeon during each task (e.g., a single cold cut). There has been growing interest in crafting techniques for discerning specific granularity from video data.

Early works for surgical procedure decomposition using classical machine-learning pipelines had limited success. CNN and recurrent neural networks (RNN) have been pivotal in enhancing workflow recognition from surgical videos and modeling spatio-temporal features. Huaulmé et al. [19] utilized CNN, RNN or both for surgical workflow recognition using Micro-Surgical Anastomose Workflow (MISAW) and found accuracy above 95%, 80%, and 60% for phases, steps, and activities, respectively. [19] Ramesh et al. [20] proposed a multi-task, multi-stage temporal convolutional network for SWR, which demonstrated improved results compared to single-task models [20]. More recently, Goodman et al. [21] developed a multitask neural network model for simultaneous, spatiotemporal analysis of hands, tools, and actions in open surgical videos. [21]

Tool usage information is another data source for understanding surgical workflow which is primarily obtained by manual labeling. Sahu et al. [22] developed a RNN to recognize tools in videos and estimate surgical phases [22]. While existing deep learning-based approaches for SWR have shown remarkable results, there is heavy reliance on large-scale labeled datasets which may be time consuming, costly, and subject to the availability of annotators with profound surgical knowledge. To address this, Shi et al. [23] validated a long-range temporal dependency-based active learning on Cholec80 video dataset and outperformed other active learning methods for SWR [23].

### Gesture recognition

Surgical gestures or “surgemes” represent the fundamental units of surgical interaction involving instruments and human tissue, such as inserting a needle, pulling a suture, or a single cut of tissue. Automatically recognizing gestures is an important element of automated activity recognition, surgeon skills assessment, surgical training, and autonomous robotic surgery systems. These gestures can serve as objective measures of surgical performance and have been found to impact surgical outcomes [24]. However, the development of automatic gesture recognition poses several challenges due to the intricacy and multi-step nature of gestures.

Gesture recognition methods are classified based on input including video, kinematics data, or both. Classical approaches for automated gesture recognition involve unsupervised learning methods such as the Hidden Markov model. Combined Markov/semi-Markov random field models employed both kinematic and video data. However, these were met with limitations accompanied by the subjectivity of manual feature extraction. Deep neural networks have proven to be a powerful tool for fine-grained surgical gesture recognition.

DiPietro et al. [25] studied gesture and maneuver recognition using RNN and found low error rates for both maneuver and gesture recognition [25]. The group demonstrated impressive accuracy for identification (AUC = 0.88) and classification (AUC = 0.87) of suturing gestures in needle-driving attempts by deep learning computer vision in patients undergoing robot-assisted radical prostatectomy (RARP) [26].

Leveraging both kinematic and video data is an essential part of accurate gesture recognition. Kiyasseh et al. [27] reported 65–97% AUC for surgical dissection gesture classification across institutions and procedures [27].

### Intraoperative assessment

While robotic surgery has achieved remarkable results across various specialties, it is undeniable that the skill of the operating surgeon plays a crucial role in surgical success. An unbiased and accurate evaluation of surgical performance is increasingly necessary in the era of AI. Traditionally, surgeon performance has been weighed through prior surgical experience or manual evaluation by experienced peers. While widely used, this technique is limited by subjectivity and labor intensity.

In the era of AI, the advent of automated performance metrics (APM) has revolutionized the evaluation of surgical performance. APMs rely on kinematic and video evaluation and serve as objective, actionable and real-time assessment tools. Combining APM with ML can produce objective assessment metrics of surgeon performance. Preliminary studies have demonstrated that APMs can differentiate expert and novice surgeon performance in clinical settings [28]. Juarez-Villalobos et al. [29] accurately classified expert (operative experience > 100 h) and non-expert (operative experience < 10 h) surgeons by time intervals during training in suturing, knot-tying, and needle-passing, three crucial surgical tasks [29]. Wang et al. [29] investigated neural networks for predicting surgical proficiency scores from video clips and achieved excellent performance with scores matching manual performance [29]. Moglia et al. utilized ensemble deep learning neural network models to identify at an early stage the acquisition rates of surgical technical proficiency of trainees. [29]

Hung et al. [31] previously used robotic surgical APMs during RARP and clinicopathological data to accurately predict continence after RARP, achieving a C-index of 0.6 via a DL model (DeepSurv) [31]. The study demonstrated that surgeons with more efficient APMs achieved higher continence rates at 3 and 6 months post-RARP. APMs were ranked higher than clinicopathological features in predicting continence. Hung et al. [32] have also demonstrated the role of ML in APM assessment to predict short-term clinical outcomes. [32]

Schuler et al. [33] utilized robotic kinematic data, surgical gesture data collected from video review, and model-integrated force sensor data in a standardized, simulation-based environment to predict surgical experience, capable of discriminating between surgeons with low or high RARP caseload with very high AUC [33].

### Surgical difficulty measurement

Surgical difficulty is a multifaceted concept in robotic-assisted surgery that encompasses not only the complexity of the tasks involved but also the cognitive workload placed on surgeons. This cognitive workload is influenced by various factors, such as the lack of tactile feedback, the need for precise communication with assistants, and the operation of multiple instruments within a limited visual field. In the context of minimally invasive surgery, despite the benefits to patients such as less postoperative pain and faster surgical wound site healing times, surgeons face significant challenges due to the physical (e.g., limited surgical field space, difficulty in reaching anatomical structures, and demands of operating on the surgical robot for extended periods of time) and cognitive demands of the procedures. AI models can help in assessing surgical difficulty. Lim et al. conducted a study where they measured physiological response patterns due to changes in workload from primary surgical tasks and multitasking requirements. They developed classification models based on these multimodal physiological signals to distinguish between the primary tasks and multitasking demands and found accuracy up to 79% [34].

### Realism in simulation

Doctors practicing on simulators before operating on patients is a crucial step that can also provide valuable data. However, the effectiveness of this practice is currently limited by the capabilities of the simulators. Many of them use basic physics, which hinders their ability to model large deformations accurately. As a result, these simulators focus on training surgeons in simplified tasks for agility rather than replicating the complexities of full surgeries. While surgeons can usually generalize the skills learned from these tasks to real clinical settings, the algorithms that aid them are only as effective as the data they receive from these simulators. Therefore, there is a significant need for more realistic simulators. Finite Element Method (FEM) is currently the benchmark for simulating deformation in soft tissue. However, its application in patient modeling is restricted due to challenges in accurately estimating parameters and its computational intensity. Accurate material parameters are crucial for precise FEM simulations. Wu et al. investigated how live data acquired during any robotic endoscopic surgical procedure may be used to correct inaccurate FEM simulation results using an open-source da Vinci Surgical System to probe a soft tissue phantom and replay the interaction in simulation. They trained a neural network to correct for the difference between the predicted mesh position and the measured point cloud and showed improved FEM results by 15–30% over various simulation parameters [35].

### Feedback optimization

**Fig. 4:**
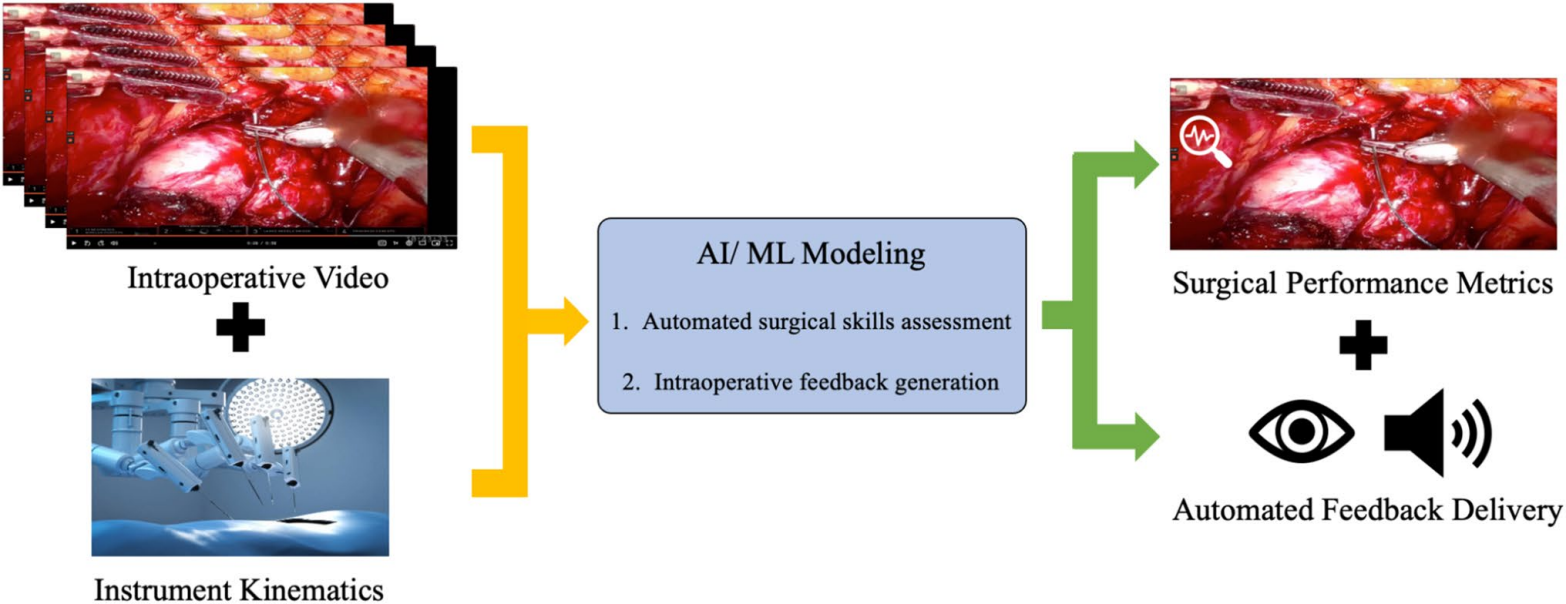
Workflow demonstrating ingestion of surgical video and kinematics data, the AI-based generation of intraoperative performance metrics and automated, tailored feedback delivery

While providing accurate and automated assessment during surgery is extremely important, a further step to enhance surgical education is the generation and delivery of targeted, high-quality intraoperative feedback. Ma et al. [36] first presented a dry lab surgical feedback exercise which showed that audio and visual feedback tailored to a trainee’s specific weaknesses improves robotic suturing skills acquisition [36]. Building on that study, Laca et al. [37] presented another robotic dissection study that used statistical modeling to categorize participants as under or overperformers. They were then given real-time audio and visual feedback which was shown to improve dissection skills in underperformers [37]. Wong et al. [38] have also developed a classification system for feedback delivered to trainees intraoperatively which lays the groundwork for determining what types of feedback are most optimal for surgical training [38]. Together, these studies are setting the stage for AI modeling that can understand a trainee’s weak points and deliver high-quality feedback for each trainee’s specific learning stage (Fig. 4**)**.

## Ethical considerations of AI in robotic surgery

### Data privacy

AI is data hungry. Developing ML models for robotic surgery requires a large amount of surgical videos, instrument kinematics, and surgeon biometrics. This requires large-scale data sharing across multiple institutions. Data privacy is a major concern, especially if compromised by cyber-attack [39]. Using a standardized way to anonymize data from data collection to data usage is critical for privacy protection [40].

### Model transparency

ML models often operate in a black box. In high-stakes environments such as the operating room, the reliability and reproducibility of intelligent-assistant algorithms are of top importance. The lack of transparency of ML models can erode trust from both surgeons and patients. For this reason, explainable AI is burgeoning, and reporting standards for AI model development are being established [41].

### Bias

Algorithms can exhibit intrinsic bias, potentially leading to healthcare disparities by perpetuating discrimination based on race, gender, or other characteristics. Bias may be rooted in datasets used for training AI models and could be mitigated by using more diverse and standardized datasets [42] or by tuning the algorithm’s training process [43].

### Accountability

Currently, it is ambiguous who would bear responsibility if a patient experiences a negative outcome due to AI-based technology. As AI algorithms are increasingly used in medical diagnosis, treatment planning, and robotic surgery, accountability is likely to be distributed among various parties. This includes the physician, the software provider, the creator of the algorithm, and possibly even the entity supplying the training data for the AI [41].

### Financial incentive

On one hand, it is important to maintain appropriate incentives for robotic manufacturers and AI developers to advance the field, aiming to improve healthcare. On the other, it is essential to prevent the unethical use of AI algorithms designed to exploit the medical system for undue profit. For instance, avoiding situations where an AI system recommends drugs, tests, or devices that are inconsistent with clinical guidelines and solely to generate profit for the parties involved [44].

Acknowledging these concerns, the U.S. is advancing AI regulations. President Biden's Executive Order on Safe, Secure, and Trustworthy AI, issued in October 2023, is a significant step. The order provides guidelines for ethical AI development, emphasizing operational security and adherence to legal standards, with a focus on ensuring safety and security in medical contexts. It also tackles potential adverse outcomes of AI like errors or biases in surgical procedures.

## Conclusion

Robotic surgery has revolutionized the way surgery is performed. As robotic surgical platforms are already highly advanced technological environments, they are a perfect place for AI models to further enhance surgical capabilities. AI models are being used to automate surgical tasks and enhance intraoperative safety. AI is also being used to enhance the field of surgical education through automated skills assessment tools and intraoperative feedback delivery. Robotic surgical AI also presents complex ethical questions that are being addressed and debated as further innovations are presented. AI implementation in robotic surgery is rapidly expanding, and we expect the future to hold more exciting enhancements.

## Data Availability

No datasets were generated or analysed during the current study.
